# Maternal and early life exposure induced AD‐ like pathophysiology and its mitigation by allicin

**DOI:** 10.1002/alz70855_102556

**Published:** 2025-12-23

**Authors:** Aluru Parithathvi

**Affiliations:** ^1^ Manipal School of Life Sciences, Manipal Academy of Higher Education, Manipal, Udupi, Karnataka, India

## Abstract

**Background:**

Alzheimer's disease (AD) is one of the most common neurodegenerative disorders characterized by amyloid plaques and neurofibrillary tangles. The increasing prevalence of AD highlights the importance of comprehending the role of major factors in disease progression. heavy metals such as lead, along with age, are key drivers of neurodegeneration. Lead, a hazardous heavy metal is adequately available in the environment and its malleable and ductile properties, increased the lead consumption. As lead can pass through placenta and blood brain barrier it is very important to study the transgenerational neurodegenerative effects of lead. Allicin, naturally obtained from *Allium sativum*, is a strong neuroprotective agent, is involved in reducing tau hyperphosphorylation and also a natural chelator due to its sulfhydryl groups.

**Method:**

In this study, Female Sprague‐Dawley rats were exposed to lead nitrate of 2mg/Kg body weight from preconception to weaning period of F1 progeny and allicin treatment of 40μg/Kg body weight. The F1 progeny were studied after 6 months of observation for behavioural histopathological and molecular changes.

**Result:**

The 6 months old F1 progeny showed significant behavioural changes attributing to anxiety and motor dysfunction between lead exposed and allicin treated groups. Similarly, histopathological studies like Nissl and H&E staining, immunohistochemistry using tau, tubulin and NeuN markers showed significant differences between lead exposed and allicin treated groups. mRNA studies of prominent AD‐specific genes like *APP, BACE1, APOE* and *PSEN1* showed significant differences between F1 progeny of lead exposed and allicin treated groups.

**Conclusion:**

The results indicate the transgenerational effect of lead in inducing AD‐like pathophysiology even at 6‐month‐old F1 progeny. Allicin treated groups showed a significant decrease in the effects indicating its chelating and neuroprotective effect. This study emphasises the transgenerational neurotoxic effect of lead, leading to early onset of major neurodegenerative disorders and its therapeutic interventions.

